# The impact of height-adjustable desks and prompts to break-up classroom sitting on adolescents' energy expenditure, adiposity markers and perceived musculoskeletal discomfort

**DOI:** 10.1371/journal.pone.0203938

**Published:** 2018-09-20

**Authors:** Ana María Contardo Ayala, Bronwyn Sudholz, Jo Salmon, David W. Dunstan, Nicola D. Ridgers, Lauren Arundell, Anna Timperio

**Affiliations:** 1 Deakin University, Institute for Physical Activity and Nutrition (IPAN), School of Exercise and Nutrition Sciences, Geelong, Australia; 2 Physical Activity Laboratory, Baker Heart and Diabetes Institute, Melbourne, Australia; 3 Mary MacKillop Institute for Health Research, Australian Catholic University, Melbourne, Australia; Leibniz Institute for Prevention Research and Epidemiology BIPS, GERMANY

## Abstract

Adolescents spend large amounts of time sitting at school. Little is known about the impact of reducing and breaking-up prolonged sitting during school lessons on adolescents’ health. This study aimed to investigate the impact of an intervention to reduce classroom sitting time on adolescents’ energy expenditure (EE; kcal/lesson), body mass index (BMI), waist circumference (WC), and musculoskeletal discomfort. A secondary school classroom was equipped with height-adjustable desks, posters promoting the health benefits of and strategies for breaking-up sitting time, and desk stickers reminding students to periodically stand up. Classroom teachers participated in a professional development session. Using a quasi-experimental design, differences between 49 participants who utilised the intervention classroom 2–5 times/week and a comparison group (39 adolescents, matched by year level and subject) who used traditional classrooms, were examined. EE, BMI and WC were objectively measured and musculoskeletal discomfort was self-reported at baseline, 4-weeks, and 17-weeks. Hierarchical linear and multilevel logistic regression-mixed models were used to examine intervention effects, adjusting for baseline values, sex and age. EE was significantly higher at 4-weeks and 17-weeks (29.4 and 37.7 kcal/lesson, respectively), BMI was higher at 4-weeks (0.34 kg/m^2^), and WC was lower at 4-weeks and 17-weeks (-3.53 and -2.64 cm, respectively) in the intervention compared to the comparison group. No intervention effect was found for musculoskeletal discomfort. Findings provide preliminary indications that these strategies may benefit health among adolescents in the short term. However, extended longer-duration trials are needed to determine longer-term health effects.

## Introduction

Recent estimates indicate that 28% of Australian adolescents are overweight or obese, and that 90% do not meet the minimum recommendation of 60 minutes of moderate- to vigorous-intensity physical activity (MVPA) every day [1]. This is consistent with most developed countries [2]. Sedentary behaviours, such as television viewing, travelling by car, and attending school lessons (while in a sitting, reclining or lying posture during waking hours) are characterised by low energy expenditure (EE< 1.5 METS) [3] and are highly prevalent in adolescents’ daily lives [1, 4]. Evidence suggests that excessive sedentary behaviours among adults have a negative impact on cardio-metabolic health independent of MVPA [5]. While there is suggestion that this relationship is attenuated when accounting for MVPA (≥ 60 min/day) [6], the levels required for this are higher than the majority prevalence rates (i.e. proportion of young people doing > 60 min/day of MVPA is 19%) [7, 8]. In younger populations, the evidence is mixed and less conclusive, and also primarily observational [9, 10]. Few studies have found that the total volume of accelerometry-measured sedentary time accumulated across the day is adversely associated with cardiovascular risk factors (i.e. elevated systolic blood pressure, triglycerides and glucose levels) [11], cardiorespiratory fitness (among girls only) [12], adiposity/obesity markers [13–15], and musculoskeletal discomfort [16]. Conversely, breaking up prolonged sitting is linked to having a lower body mass index (BMI) and a reduced risk of being overweight [17], enhanced fitness [18], and lower diastolic blood pressure [19] among adolescents. However, this evidence is primarily cross-sectional, and longitudinal observational findings are inconsistent [20]. Experimental evidence, using objective measures of sitting, is needed to determine whether reductions and interruptions to prolonged periods of sitting can benefit adolescent health.

Given the high volume of sitting that usually occurs in the school classroom [21], this setting has been identified as an important environment for interventions aimed at reducing and breaking up adolescents’ prolonged sitting time and preventing the associated deleterious health consequences [22]. During school hours (which equates to approximately 6.5 hours per day, five days a week, 40 weeks per year in Australia), adolescents can spend up to 80% of their time sitting (approximately 26 hours per week) [23, 24], most notably during classroom lesson times. Despite this, most school-based interventions to date have focused on increasing physical activity during recess, lunch and physical education, rather than targeting class time sitting [25]. Identifying strategies to reduce prolonged sitting during classroom lessons may therefore be beneficial for adolescents’ health.

Among primary (elementary) school children, the introduction of height-adjustable desks that allow students to work either in sitting or standing postures has received recent attention, with some studies showing a positive impact on sitting time and light-intensity physical activity (i.e. steeping/standing) [26–30], energy expenditure (EE) [31–33], and academic engagement [34]. However, findings regarding their impact on other indicators of children’s health, such as musculoskeletal discomfort [30], BMI [29, 30, 35], waist circumference (WC), and blood pressure [30] are mixed. While these studies used a similar environmental strategy (i.e. replacing traditional seated desks with height-adjustable or stand-biased desks), they varied by the intervention length (4 weeks [28] to 2 years [35]), sample size (8 [29] to 337 [33]), and study design (within subjects vs. randomised/non-randomised controlled). This makes comparing results across studies difficult.

To the best of our knowledge, only two pilot studies have tested the impact of using height-adjustable (n = 43; mean age 13.7 years; 7 week intervention length) [23] and stand-biased (i.e. fixed height, adjusted to appropriated user’s level) desks (n = 34; mean age 14.6 years; 27 week intervention length) [36] among adolescents in secondary (high) school. These studies found that compared to the traditional classroom setting, adolescents reduced their class time sitting [23], and improved executive function and working memory [36]. However, some adolescents (50%) reported adverse effects such as leg or back pain associated with increased standing [23]. No studies have examined the potential impact of height-adjustable desks and other strategies to reduce and break up sitting in the classroom on adolescents’ health outcomes such as EE, BMI and WC. Further, enhanced benefits and diminished musculoskeletal discomfort may be possible through additional supportive strategies to supplement the introduction of height-adjustable desks and instructions on how to correctly adjust the desks, respectively [21].

The aim of this study was to investigate the impact of introducing height-adjustable desks in a secondary school classroom, accompanied by additional supportive prompts that encouraged breaking up sitting time, on EE, BMI, WC and perceived musculoskeletal health among adolescents.

## Methods

### Study design and participants

A quasi-experimental intervention trial was conducted in one government secondary school in Melbourne, Australia, across two school terms (June to November 2015: winter and spring terms). Approval was received from Deakin University Human Ethics Advisory Group (Health) (HEAG-H 93_2014) and the Department of Education and Training (2014_002402).

After obtaining written informed consent from the school Principal, one classroom was assigned as the intervention classroom (due to space availability). Students from Years 7, 10 and 11 (mean age 14.8 ±1.7 years, age range 12 to 17 years) with timetabled classes in the intervention classroom and a comparison (control) group of students (i.e. matched on year level and school subject) who only used the traditional ‘seated’ classrooms were invited to attend a recruitment session at the school (May 2015). This session involved a brief oral presentation by the research team explaining the study aims, and the distribution of plain language statements and consent forms. Parents/carers/guardians provided consent on behalf of their child to participate in core elements of the evaluation (i.e. a student survey). Parents could also provide additional optional consent for their child to wear a SenseWear Armband (Body Media, Inc, USA) and have anthropometric measures taken. Eligible teachers (intervention and control classroom teachers matched on subject and year) were also invited by the research team to participate in the study. Data collection took place at baseline (week 0), week 4 and week 17.

### Procedures

After baseline data collection, a one-hour professional development session was held for the teachers timetabled to use the intervention classroom. This session included a presentation (1.5 hrs) by the research team outlining the study purpose and supportive prompts (outlined in detail below). Teachers were provided with the printed manual used for this information session. They were asked to inform their students of the health benefits of regularly breaking up prolonged sitting time, assist with directing students to the classroom posters, and encourage them to break up classroom sitting every 15 minutes with standing for at least 2 minutes (Note: the 15-minute period was chosen based on cross-sectional and experimental studies among children [37] and adults [38], that suggested that periods longer than 15 minutes may have negative health consequences). Following the professional development session, the intervention components were installed in the classroom.

### Intervention components

#### Height-adjustable desks

Traditional classroom furniture in the intervention classroom was replaced with height-adjustable desks (Learnfit, Ergotron Inc., Minnesota, USA) and lab stools (Furnware Bodyfurn Lab stool, New Zealand) for every student and the teacher.

#### Additional supportive prompts

The supportive prompts used in the intervention focused on four key messages: (1) the health impacts of excessive sitting time and the potential health benefits of breaking up prolonged sitting time; (2) breaking up sitting every 15 minutes by standing for at least two minutes; (3) tips and strategies on how to reduce and break up classroom sitting, (e.g. stand-up when using computer or tablet, reading, group work); and (4) how to appropriately use and adjust the desks, the correct posture while sitting and standing, how to shift the balance between the feet while standing, and how to stretch upper and lower limbs muscles.

The supportive prompts were described to the teachers during their professional development session. Three informative posters showed these prompts and were displayed on the walls of the intervention classroom (size A2). Stickers prompting students to break up their classroom sitting every 15 minutes were also placed on the upper left-hand corner of each desk as a visual prompt.

The comparison group (students and teachers) followed standard pedagogical (usual) practice in classrooms using traditional ‘seated’ furniture.

### Measures

All participants in the evaluation (intervention and comparison groups) undertook measurements concurrently at baseline (week 0) and at weeks 4 and 17, with the exception of the questionnaire that was administered at baseline and 17-weeks to reduce participant burden. All assessments were taken at the school by trained project staff.

#### Energy expenditure (EE)

EE was measured using a SenseWear Armband (BodyMedia, Inc., Pittsburgh, USA) at each time point. This device is worn on the upper left arm and integrates accelerometry with skin sensors that measure heat flux, galvanic skin responses, and near body ambient temperature [39]. Sensewear Armband is a valid and reliable tool for estimating EE [39, 40]. Participants wore the device during waking hours for five consecutive school days (it was optional to wear the monitor at night) at each of the three assessment time points [41]. Information about the correct wear and care of the monitors was provided to the students. Each monitor was configured with the adolescent’s sex, age, stature, body mass, and handedness using the proprietary software (SenseWear software v 7.0, BodyMedia).

At the conclusion of data collection, data were downloaded and processed into 1-min epochs using algorithms provided within the proprietary software. The epoch length is set by the manufacturer and cannot be altered. These data were then analysed using a customised Microsoft Excel macro. The SenseWear directly identifies periods of non-wear via the skin sensors [41]. The start and end times of periods of interest (i.e. lessons in the intervention and comparison classrooms, 57 minutes per lesson) were identified using the school timetable. Participating students attended lessons in the intervention classroom approximately two to five times per week. EE data (i.e. kcal/lesson) during these periods were extracted by the macro for analysis. Data were considered valid and included in the analysis if the participant had worn the monitor for at least 50% of the lesson and was recorded as present at school, in accordance with previous research [30, 42]. EE was calculated for each valid lesson in the intervention classroom, averaged across all valid lessons, and standardised according to total lesson wear time and multiplied by lesson length. Data from the comparison group for matched lessons were analysed in the same way.

#### Anthropometric measures

Adolescents’ stature was measured to the nearest 0.1 cm using portable stadiometers (Seca, 0123), and body mass was measured to the nearest 0.05 kg using portable calibrated electronic scales (Tanita, InnerScan 50) at each time point. WC was assessed using a flexible steel tape at the umbilicus. Two measurements were taken for stature, body mass, and waist circumference. Where a discrepancy of >0.5 cm or >0.5 kg was apparent, a third measurement was taken and the average of each was used. BMI (kg/m^2^) was calculated based on stature and body mass and participants were categorised according to the International Obesity Task Force definitions of healthy weight or overweight/obese [43].

#### Demographics

A brief questionnaire was administered to students by research staff at baseline to obtain socio-demographic characteristics (e.g. age, sex).

#### Musculoskeletal discomfort

To assess the location of any musculoskeletal discomfort, the modified Nordic Musculoskeletal Questionnaire was administered at baseline and week 17, which has been used and validated in children previously [27]. Participants were asked to report if, during classroom lessons over the last seven days, they had experienced any muscle, joint or bone discomfort (yes/no). If the answer was ‘yes’, students were asked to select the areas on a diagram of the body (i.e. anterior and posterior view) and then mark the intensity of the discomfort using a 5-point visual analogue discomfort scale ranging from (1) no discomfort, to (5) unbearable discomfort. Questions related to the perception of musculoskeletal discomfort were grouped into three body sites: 1) upper limbs (shoulder, elbow, forearm, upper-arm, wrist/hand), 2) lower limbs (buttock/hip, thigh, knee, calf, ankle and foot), and 3) back/spine (neck, upper and lower back). Each body site was coded as 1 (yes) or 0 (no).

### Statistical analyses

Statistical analyses were conducted using Stata 14.0 (StataCorp LP., College Station, TX, USA). Statistical significance was set at *p* <0.05. Descriptive statistics were used to describe the sample and percentage of participants who provided valid data at each time point. Independent *t*-tests and Pearson’s chi-square tests were used to compare groups at baseline. Hierarchical linear mixed models were used to examine intervention effects on EE, WC and BMI at 4 and 17 weeks. Multilevel mixed-effects logistic regression models were used to examine intervention effects on self-reported discomfort in the upper limbs, back/spine and lower limbs. For all models, the unit of analysis (students) was nested within clusters (intervention or comparison groups), and baseline values, age and sex were adjusted for.

## Results

Overall, 105 adolescents (i.e. 62% response rate) provided parental consent to complete the survey and a subsample of 93 adolescents provided additional consent for the SenseWear and anthropometric assessments. Table 1 reports participants’ characteristics for the intervention and comparison groups at baseline and the percentage of participants that provided valid data at each of the three time points. With the exception of participants in the intervention group being significantly older than the comparison group (15.3 vs. 14.4 years old, respectively), there were no significant between-group differences at baseline.

**Table 1:** Baseline characteristics of participants in the intervention and comparison groups (mean ± standard deviation [SD], or percentages) and percentage of participants who provided valid data at 4 and 17 weeks.

	Intervention (n = 49)	Comparison (n = 39)	*p*-Value*
**Age** (years)	15.3 (1.7)	14.4 (1.7)	**0.008**
**Girls** (%)	38.8	48.7	0.350
**EE** (kcal/lesson) (mean, SD)	112.0 (34.9)	110.3 (47.4)	0.840
**WC** (cm) (mean, SD)	75.5 (7.9)	72.7 (9.2)	0.137
**BMI** (kg/m^2^) (mean, SD)	20.6 (1.9)	20.9 (4.5)	0.636
**BMI categories** (%)			0.119
Normal weight	95.6	84.2	
Overweight	4.4	7.9	
Obese	0	7.9	
**Presence ofmusculoskeletal discomfort** (%)			
Upper limbs	28	26	0.846
Back/spine	36	26	0.313
Lower limbs	32	21	0.256
**Valid Data** (%)			
SenseWear			
Baseline	90.4	90.2	0.974
4-Week	80.8	82.9	0.794
17-Week	90.4	70.7	**0.014**
Anthropometry			
Baseline	92.3	97.6	0.260
4-Week	84.6	70.7	0.105
17-Week	90.4	78.0	0.097
Musculoskeletal			
Baseline	90.9	84	0.283
17-Week	90.9	66	**0.001**

(*) Significant differences (p<0.05) are highlighted in **bold**.

Abbreviations: EE, energy expenditure; WC, waist circumference; BMI, body mass index

### Impact of the intervention on energy expenditure (EE) and anthropometric measures

The 4- and 17-week changes from baseline and intervention effects on EE and anthropometric measures are shown in Table 2. Compared to the comparison group, the intervention group expended significantly more energy during a typical lesson at 4 and 17 weeks. Relative to the comparison group, WC was significantly lower in the intervention group at 4 and 17 weeks. While BMI was significantly higher at 4 weeks, no significant intervention effect on BMI was observed at 17 weeks.

**Table 2:** Changes in energy expenditure (EE), waist circumference (WC), and body mass index (BMI) from baseline for the intervention and comparison groups and intervention effects between groups.

	4-week change from baseline			17-week change from baseline		
	Intervention Mean (SE)	Comparison Mean (SE)	Intervention effects (95% CI)	Intervention Mean (SE)	Comparison Mean (SE)	Intervention effects (95% CI)
**EE (kcal/lesson^∞^)**	15.3 (6.2)*	-14.1 (7.0)*	**29.4 (11.1, 47.7)** ^†^	34.8 (6.5)*	-2.88 (8.4)	**37.7 (16.9, 58.5)** ^†^
**WC (cm)**	-1.6 (0.9)	2.0 (1)	**-3.5 (-6.3, -0.8)** ^†^	-0.32 (0.7)	2.33 (0.9)	**-2.6 (-5.0, -0.3)** ^†^
**BMI (kg/m^2^)**	0.3 (0.1)*	-0.0 (0.1)	**0.3 (0.1, 0.6)** ^†^	0.5 (0.1)*	0.2 (0.1)	0.3 (-0.1, 0.6)

^**∞**^ EE standardised for a 57 minute lesson

* *P* < 0.05 within-group difference from baseline

† *P* < 0.05 between-group difference from baseline, are highlighted in **bold**.

Note: All models were adjusted for baseline values, age and sex.

Abbreviations: BMI = body mass index; cm = centimetres; EE = energy expenditure; SE = standard error; WC = waist circumference; 95% CI: 95% confidence interval.

### Impact of the intervention on self-reported musculoskeletal discomfort

No significant intervention effects were found in relation to musculoskeletal discomfort after the 17-week intervention period (Table 3).

**Table 3:** Odds ratios (95% confidence internal [95%CI]) of reporting musculoskeletal discomfort at 17 weeks among the intervention group relative to the control group.

	Upper limbs OR (95% CI)	Back OR (95% CI)	Lower limbs OR (95% CI)
**Control group** (Ref)	1.0	1.0	1.0
**Intervention group**	0.76 (0.26, 2.21)	1.0 (0.34, 2.90)	1.94 (0.62, 6.04)

Note: All models were adjusted for baseline values.

Abbreviations: OR: Odds Ratio; 95% CI: 95% confidence interval.

## Discussion

This quasi-experimental study aimed to determine the impact of introducing height-adjustable desks in a classroom, accompanied by additional supportive prompts encouraging breaking-up sitting time, on EE, adiposity markers, and perceived musculoskeletal discomfort among adolescents. The results indicate that relative to the comparison group, the intervention group expended significantly more energy, had a lower WC and higher BMI (4 weeks only), but did not report more musculoskeletal discomfort. After 17 weeks, adolescents who used the intervention classroom expended approximately 38 kcal more energy per lesson compared to those who used the traditional classrooms. While it is acknowledged that this is a small increase in EE, this could have an important effect on weight gain prevention if maintained in the long-term. Notwithstanding the numerous status quo assumptions (e.g. energy balance remains fixed and students have access to the height-adjustable desks for all their lessons throughout the school year), if this effect was to be extrapolated to a school week, this could equate to 950 kcal/week. Further research with larger samples and longer follow-up periods are necessary to determine if such changes would have clinical significance for weight gain prevention or other health consequences.

The positive impact on EE is consistent with previous studies using height-adjustable desks among primary/elementary school children, as assessed by BodyBugg (n = 19, aged 6 to 8 years) [32] and SenseWear (n = 337, mean age 8.5 years) [33] monitors. These studies showed increases in EE of approximately 16 kcal/hour (compared to baseline) and 9.6 kcal/hour (relative to control group), respectively. In the current study, the mean difference in EE over time relative to the comparison group (approximately 40 kcal/hour) was greater than what has been observed in these previous studies, suggesting that introducing height-adjustable desks and prompts into classrooms could have a greater impact on EE among adolescents than among children. Although it was expected that adolescents would have greater EE than children as a result of greater body mass and size, the larger magnitude of change in adolescents’ EE could also be because secondary schools are usually associated with more sedentary/sitting time compared to primary schools (70 vs 80%, respectively [21, 23]) where children may stand/move more frequently within the classroom [44]. Therefore classroom-based strategies that aim to reduce and/or break up prolonged sitting time may have greater benefits for adolescents. It is important to note that the intervention effects on EE were larger at 17 weeks compared to 4 weeks, which may indicate that the prompts helped to maintain changes over time. However, further research, using other process measures to assess the impact of prompts, within diverse secondary schools over longer periods is needed to test this further.

At 17 weeks, WC was 2.6 cm lower among participants in the intervention group, compared with the comparison group. This contrasts a previous 8-month primary school study (n = 48, mean age 11.6 years) that showed no significant intervention effects on intervention children’s WC (+1.53 cm difference) relative to the control group [30]. The results of the current study may suggest small changes in the central (abdominal) mass after the intervention, which could be indicative of an overall fat mass reduction. Although the current findings are positive, objective measurements (e.g. Dual-energy X-ray absorptiometry) should be used to determine any changes in total and regional body composition after using height-adjustable desks over a prolonged period of time.

Despite the differences in WC, no significant intervention effects were found for BMI in the current study at 17 weeks, which is consistent with a previous 8-month study in primary-aged children [30]. In contrast, longitudinal evidence from a randomised controlled trial among primary school students (n = 380, mean age 8.8 years) showed positive changes on BMI percentile (5.24 percentile change) after 2 years of exposure to stand-biased desks in classrooms, compared to children using traditional seated classrooms [35]. It is possible that interventions longer than 17 weeks are needed to elicit an effect on BMI among adolescents. However, it is also possible that the lack of findings in relation to BMI in the current study could be explained by pubertal or growth-related differences (e.g. BMI change/increase with age) among adolescents. A limitation of this study was that pubertal status was not assessed, which could be reflected in the higher BMI at 4 weeks. In addition, as described in the introduction, sedentary behaviour may not be independently associated with adiposity, but is perhaps an indicator or facilitator of other unhealthy lifestyle habits such as a poor diet [7]. It is also important to consider that just 70% of the comparison group provided valid anthropometric measures at 4 weeks compared to 85% of the intervention group. Although there were no differences in BMI at baseline, there may have been attrition bias at the 4-week time point. Additionally, BMI is less sensitive than WC as an indicator of adiposity, and therefore, less sensitive to measure individual change [45].

Up to one-third (36%) of participants reported some aspect of musculoskeletal discomfort at baseline. The null effect of the intervention on adolescents’ perceived musculoskeletal discomfort can be viewed as a positive outcome in that students reported no adverse effects when using a height-adjustable desk. In a previous pilot with secondary school students, there was an increase in the number of students reporting discomfort in the back or legs after using height-adjustable desks for 7-weeks [23]. This may have been because students were still adjusting to periods of standing during class and/or may have required posture recommendations to avoid muscle discomfort [21], however, for this study the adjusting period was not assessed. In the current study, adolescents and teachers received instructions and training about correct adjustment of the workstation to students’ height, as well as recommendations about optimal sitting and standing posture during lessons, as depicted on the classroom posters. Further, teachers and students were made aware of the evidence that shows frequent postural transitions, rather than standing for long periods, are associated with potential acute benefits [38]. These approaches may have helped to prevent musculoskeletal discomfort while using the desks. However, more objective posture measures (e.g. direct observation) are needed to determine sitting/standing posture while using the desks to provide specific posture recommendations to prevent musculoskeletal discomfort in future studies.

The main strengths of this study included the unique implementation of the intervention in a secondary school setting, the use of additional strategies to support the use of height-adjustable desks in the classroom, inclusion of a mid-point assessment, and the use of an objective measure of EE capable of detecting small changes during the classroom/lesson of interest. This study also had some limitations, including a non-randomised design with the comparison group being from the same school. Although this increased the risk of contamination, energy expenditure decreased rather than increased in the comparison group and there were no other significant differences over time for this group. Participants in the intervention group were older than those in the comparison group, which could be considered a limitation. While all models adjusted statistically for age, this may have affected some of the findings. Additional limitations included a small sample size, the limited exposure to the desks (2–5 lessons per week) which may have diminished the impact of the intervention, the short duration of the intervention, the absence of assessment of pubertal status, differences in the size of intervention and comparison groups, and variability in the number of participants within each group who had valid data (i.e. Sensewear Armband and musculoskeletal data at 17 weeks), which could cause attrition bias. Future research should establish the long-term heath impact of installing height-adjustable desks in secondary schools, and elucidate whether such benefits can be sustained over time. There is little evidence investigating the impact of breaking-up sitting using height-adjustable desks and pedagogical strategies on academic outcomes [21, 46]. However, classroom-based physical activity may have a positive impact on academic related outcomes (i.e. on-task behaviour and academic achievement) [47–49], therefore future research should explore the potential additional benefits of height-adjustable desks on cognitive outcomes, academic achievement and classroom behaviour.

## Conclusion

The introduction of height-adjustable desks and supportive prompts in a secondary school classroom had a positive, albeit small, impact on EE during lessons and waist circumference at 4 and 17 weeks relative to a comparison group, and did not negatively impact perceived musculoskeletal discomfort. The findings suggest that such intervention strategies may contribute to health benefits among adolescents in the short term, but studies with greater weekly exposure to these strategies are needed. Furthermore, such strategies may help create lifelong healthy behaviours by raising awareness of the importance of breaking up sitting. Future research with larger samples over longer periods are needed to support these findings.
